# Electrolyte and acid-base imbalance in severe COVID-19

**DOI:** 10.1530/EC-21-0265

**Published:** 2021-06-22

**Authors:** Anna Sjöström, Susanne Rysz, Henrik Sjöström, Charlotte Höybye

**Affiliations:** 1 Department of Molecular Medicine and Surgery, Karolinska Institutet, Stockholm, Sweden; 2 Department of Clinical Chemistry, Karolinska University Hospital, Stockholm, Sweden; 3 Function Perioperative Medicine and Intensive Care, Karolinska University Hospital, Stockholm, Sweden; 4 Department of Medicine Solna, Karolinska Institutet, Stockholm, Sweden; 5 Department of Clinical Neuroscience, Karolinska Institutet, Stockholm, Sweden; 6 Center for Neurology, Academic Specialist Center, Stockholm, Sweden; 7 Department of Endocrinology, Karolinska University Hospital, Stockholm, Sweden

**Keywords:** electrolytes, COVID-19, hyperaldosteronism, RAAS, hypernatremia

## Abstract

Acute systemic diseases, such as severe infections, can lead to electrolyte and acid-base alterations. To study the presence of electrolyte imbalance in severe COVID-19, we investigated the frequency and consequences of changes in electrolyte and acid-base patterns over time. We performed a retrospective cohort study including 406 patients with severe COVID-19. Levels of electrolytes, base excess, pH, serum osmolality, and hematocrit, the first 2 weeks of hospitalization, were collected daily from the laboratory database and clinical data from patients’ medical records. We found that hyponatremia was present in 57% of the patients at admission and 2% in hypernatremia. However, within 2 weeks of hospitalization 42% of the patients developed hypernatremia, more frequently in critically ill patients. Lower levels of sodium and potassium during admission were associated with the need for mechanical ventilation. Decreased pH at admission was associated with both death and the need for mechanical ventilation. Hypernatremia in the ICU was combined with rising base excess and a higher pH. In the group without intensive care, potassium levels were significantly lower in the patients with severe hypernatremia. Presence of hypernatremia during the first 2 weeks of hospitalization was associated with 3.942 (95% CI 2.269–6.851) times higher odds of death. In summary, hypernatremia was common and associated with longer hospital stay and a higher risk of death, suggesting that the dynamics of sodium are an important indicator of severity in COVID-19.

## Introduction

Electrolyte and acid-base imbalance are common in many types of severe diseases. In the SARS-Cov-2 pandemic, the combination of respiratory failure and metabolic changes due to organ failure results in unpredictable electrolyte and acid-base patterns.

Hyponatremia in severely ill patients in the intensive care unit (ICU) is a known complication of somatic diseases such as pneumonia and heart failure (1). Several reports of hyponatremia at admission in COVID-19 patients all indicate that the low initial levels of plasma sodium could be a risk indicator of severe disease and mortality (2, 3, 4, 5). Syndrome of inappropriate secretion of antidiuretic hormone (SIADH) is a common cause of hyponatremia in viral pneumonia, and there are multiple case reports of this neuroendocrinological disturbance in COVID-19 patients (6, 7, 8, 9, 10). In SIADH, there is an increased release of AVP, from the posterior lobe of the pituitary, despite normal or low serum osmolality. Increased levels of AVP lead to hyponatremia due to excessive inflow of water through aquaporins in the tubules of the kidney (11).

Hypernatremia is not uncommon in the ICU, and previous studies have reported a frequency of 4.3–15.8% depending on the type of ICU (medical/surgical) and the definition of hypernatremia as above 145 or 150 mmol/L) (12, 13, 14, 15, 16, 17, 18). It can be a serious condition associated with extended hospital stay and increased mortality (19). The causes of ICU-related hypernatremia are often considered iatrogenic due to saline infusions and the patient’s loss of autonomy and inability to drink when thirsty (13). Complications associated with hypernatremia and hyperosmolality include dehydration, hyperventilation, muscle cramps, rhabdomyolysis, and brain shrinkage due to dehydration (20).

A publication of a small cohort of patients, from the first phase of the pandemic, with severe COVID-19, indicates the importance of therapy-resistant hypernatremia which prolonged the total hospital stay (21). In a recent study, hypernatremia during hospitalization for COVID-19 was found to be associated with both the need for mechanical ventilation and mortality. Sodium level was suggested to be used as part of a risk stratification disease severity (22). Although these studies have increased the knowledge of hypernatremia and COVID-19, it is still limited, and further investigations of electrolyte imbalance and the pathophysiology behind are needed.

Regarding other electrolytes and acid-base biomarkers, there are only a few studies published. Low levels of potassium during admission have been associated with poor prognosis and severe disease, but little is known of the dynamics over time in COVID-19 (23, 24). Increased levels of urinary potassium have been found in critically ill patients with COVID-19 (25). Chloride homeostasis has been briefly discussed in previous studies without finding any prognostic value of the level during admission (26). In patients admitted to the ICU, alkalosis has been seen in arterial blood (27).

The reported deviations in electrolytes during COVID-19 can affect treatment, length of hospital stay, and mortality. Patients with severe COVID-19 are often hospitalized over long periods, and further characterization of electrolyte and acid-base levels over time are, therefore, of great interest.

The aim of this study was to examine the dynamics of routine plasma electrolytes and acid-base biomarkers in patients with severe COVID-19 and if they were associated with the need for mechanical ventilation and mortality. Electrolyte levels together with base excess, pH, and hematocrit were also evaluated.

## Materials and methods

The study was a retrospective observational cohort study of 428 consecutive patients with severe COVID-19 admitted to Karolinska University Hospitals (Karolinska) Solna and Huddinge, between March 5 and April 20, 2020. The study was approved by The Swedish Ethical Review Authority (SERA), Regional Board of Linköping (N 2020-01752). Due to the retrospective nature of this project, the need for informed consent was waived in accordance with the approval from SERA. The study was conducted in accordance with all the relevant guidelines and regulations.

Inclusion criteria were positive RT-PCR for SARS-Cov2, admission to a hospital connected to the Karolinska laboratory, and being assessed as having severe disease defined by SaO2 < 94% on room air at sea level (28, 29). Patients were followed, by reviewing medical records, until discharged from the hospital or deceased. Last day of follow-up was June 30, 2020.

Patients transferred to a hospital in another region, before they recovered, were excluded.

Descriptive data on age, gender, BMI, weight, comorbidities (medically treated diabetes mellitus type 2 (DM II), hypertension, and obstructive pulmonary disease (OPD)) were retrieved together with information on the day of hospitalization and admittance to ICU, need for intubation and length of mechanical ventilation, presence of acute kidney injury (AKI) and continuous renal replacement therapy (CRRT), death and total hospital stay.

### Outcomes

Patients were defined as discharged when dismissed from inpatient care to their homes or a rehabilitation facility. Information on mortality was retrieved from the medical records from admittance to the hospital until the last day of follow-up. A critically ill patient, including in the ICU group, was assessed by a specialist in anesthesiology to be in need of intensive care. Patients included in the ward group were never admitted to the ICU. AKI was classified as present when noted in the medical records or by ICD code. Three groups were defined according to the patients’ peak level of sodium; non-hypernatremia (<145 mmol/L), moderate hypernatremia (145–149 mmol/L), and severe hypernatremia (>149 mmol/L). Presence of hyponatremia, defined as plasma sodium < 137 mmol/L, was also recorded.

Laboratory parameters were collected from the Karolinska laboratory database. Information on levels of plasma sodium, potassium, and serum osmolality as well as blood gas analysis of arterial chloride, base excess (BE), and pH was recorded from the day of admittance and onwards daily, when measured, until 2 weeks had passed. If there were several measurements of a parameter the same day, the result closest to 8.00 AM was recorded. Information on hematocrit and plasma creatinine levels at admission and at the day of peak sodium level was retrieved. For the non-hypernatremia group, the median day of peak sodium in the hypernatremia group was used for extraction of the second hematocrit and plasma creatinine values. For more information on laboratory equipment and methods, see Supplementary Table 1 (see section on supplementary materials given at the end of this article).

**Figure 1:**
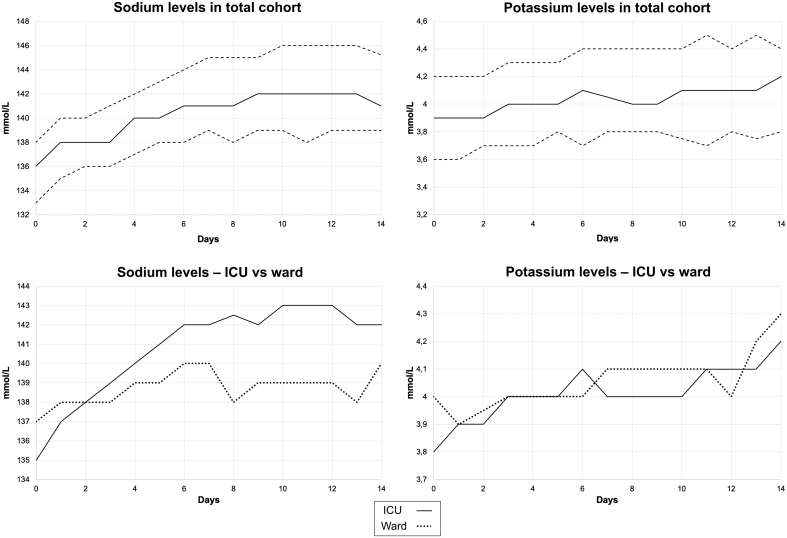
Sodium and potassium dynamics over time in all patients. Dynamic pattern of laboratory parameters (sodium and potassium) of 406 patients with severe COVID-19 during the first 2 weeks of hospitalization. Upper row is median sodium and potassium levels (solid lines) of the total cohort with upper and lower quartiles (broken lines); Bottom row is median sodium and potassium levels of patients with or without the need of intensive care.

Study size was finalized at 406 patients after exclusion of 22 patients due to loss to follow-up. The number of patients with relevant blood tests at the respective time points, regarding Fig. 1, is shown in a Supplementary Table 2.

### Statistical analysis

Statistical analysis was performed using Excel (Microsoft office 365, Microsoft Corp.) and SPSS (version 26.0, IBM Corp.). Normality of data was assessed using Shapiro–Wilk test. Pearson’s *χ*^2^ test was used to evaluate possible differences in distributions between groups. Binary logistic regression was used to investigate the effect of clinical variables on the risk of death, and the risk of need for mechanical ventilation. Medians at each time point were calculated from existing laboratory results, and no interpolation of data was performed. Kruskal–Wallis test was used for assessments of group differences and Mann–Whitney *U*-test for pairwise comparisons. In critically ill patients, electrolyte levels, pH, and base excess were examined on days 0, 4, 8, and 12. Wilcoxon signed ranks test was used to evaluate changes in plasma creatinine and hematocrit between admission and day of peak sodium within the groups. A *P*-value <0.05 was considered significant.

## Results

In our total cohort of 406 patients with severe COVID-19, hyponatremia during admission was seen in 219 (57%) patients, and during 2-week study period, 299 (74%) patients had at least one sodium result below 137 mmol/L. Over time, sodium in plasma increased to the level of hypernatremia in 170 patients (42%) and 61 (15% of the total cohort) of these patients had peak levels of 150 mmol/L or above.

### Descriptive statistics of the total cohort and subgroups

Median age of the total cohort was 59 years, the majority were males (75%), median BMI was 29 kg/m^2^, and the most common comorbidities were hypertension (39%) and diabetes mellitus type 2 (30%), see Table 1. No patients received treatment with dexamethasone.

The cohort was further divided into three groups, depending on the development of hypernatremia and its severity, and the descriptive variables are presented in each group. No differences in the distribution of descriptive variables were found between the groups with moderate hypernatremia, severe hypernatremia, or normonatremia (Table 1). There was a smaller proportion of patients with treated hypertension and a larger proportion of treated DM II in the group with severe hypernatremia compared to the other groups, but not statistically significant with *P* = 0.171 and *P* = 0.064, respectively.

**Table 1:** Descriptive admission data of 406 patients with severe COVID-19 presented as medians with interquartile range or count with percentage.

	All	Non-hypernatremia (<145 mmol/L)	Moderate hypernatremia 145–149 mmol/L	Severe hypernatremia > 149 mmol/L	*p*
Patients, *n* (%)	406	236 (58)	109 (27)	61 (15)	
Age, years (IQR)	59 (50–66)	60 (46–67)	59 (52–64)	58 (52–67)	0.525
Male sex, *n* (%)	304 (75)	171 (73)	82 (75)	51 (84)	0.201
BMI (IQR)	29 (26–33)	28 (26–33)	30 (27–33)	29 (26–31)	0.107
Weight, kg (IQR)	87 (76–98)	86 (73–98)	90 (79–100)	85 (76–94)	0.228
Hypertension, *n* (%)	157 (39)	96 (41)	44 (40)	17 (28)	0.171
Diabetes mellitus type 2, *n* (%)	115 (30)	58 (25)	33 (30)	24 (39)	0.064
OPD, *n* (%)	73 (19)	42 (18)	24 (22)	7 (11)	0.228

Another subgroup analysis was performed dividing the total cohort into two groups depending on whether the patient was stable enough to only be treated at standard level of care (the ward group) or if the patient was in need of intensive care (the ICU group) during the time in hospital. The two groups were further divided into three groups depending on the development of hypernatremia, as described above. Less patients with treated hypertension were found in the hypernatremia groups in the ICU group compared to the non-hypernatremia group (*P* = 0.032), and a higher proportion of patients with DM II was seen in the hypernatremia groups in the ward group (*P* < 0.001). There were no other significant differences found in the subgroup analysis regarding the descriptive variables of Table 1.

### Statistics of outcome variables of the total cohort and groups depending on peak sodium level

During the study period, 55% were in need of ICU care, 47% of the patients received mechanical ventilation, 26% developed acute kidney injury (AKI), and 21% did not survive (Table 2). When divided into groups based on peak sodium, there was a significantly larger proportion of patients in need of intensive care, mechanical ventilation, dialysis as well as deceased in the hypernatremia groups compared to the non-hypernatremia group.

**Table 2:** Outcome data of 406 patients with severe COVID-19.

	All	Non-hypernatremia (<145 mmol/L)	Hypernatremia 145–149 mmol/L	Hypernatremia > 149 mmol/L	*p*
Need of intensive care, *n* (%)	223 (55)	77 (33)	93 (85)	53 (87)	< 0.001
Need of mechanical ventilation, *n* (%)	191 (47)	47 (20)	91 (83)	53 (87)	< 0.001
Median time on ventilator^a^ (IQR)	14 days (8–22)	12 days (4–21)	14 days (8–21)	15 days (10–24)	0.218
Median time in ICU^b^ (IQR)	14 days (8–22)	8 days (3–18)	16 days (9–24)	17 days (11–26)	< 0.001
Median hospital stay (IQR)	15 days (8–26)	10 days (6–17)	22 days (15–35)	21 days (17–39)	< 0.001
Acute kidney injury, *n* (%)	106 (26)	28 (12)	49 (45)	29 (48)	< 0.001
Need of dialysis, *n* (%)	51 (13)	17 (7)	25 (23)	9 (15)	< 0.001
Mortality, *n* (%)	87 (21)	29 (12)	38 (35)	20 (33)	< 0.001

^a^Only applicable for the intubated patients; ^b^Only applicable for the 223 critically ill patients.

### Effects of sodium, potassium, chloride, and pH during admission on the likelihood of death and need for mechanical ventilation

A decrease in pH levels during admission by 0.1 did significantly increase the odds of death by 2.374 (95% CI 1.398–4.033) (*P* = 0.001), using binary logistic regression and controlling for age, gender, and presence of diabetes mellitus type 2, hypertension, and OPD. There was no association with death found for admission levels of sodium, potassium, and chloride, (*P* = 0.626), (*P* = 0.820), and (*P* = 0.866), respectively.

A decrease in sodium or potassium level during admission, by 1 mmol/L, increased the odds of need for mechanical ventilation by 1.153 (95% CI 1.040–1.280) and 2.752 (95% CI 1.315–5.763), respectively (*P* = 0.007 for both). A decrease in pH level during admission by 0.1 increased the odds of need for mechanical ventilation with 2.864 (95% CI 1.440–5.697) (*P* = 0.003). There was no association with mechanical ventilation found for admission levels of chloride (*P* = 0.343).

### Laboratory parameter dynamics over time

Regarding the whole cohort, the dynamics of sodium and potassium levels over the first 2 weeks of hospitalization are presented in Fig. 1. For base excess, pH, and chloride levels, there were too much missing data in the ward population after the admission date, and results are only presented from levels during admission. Results of serum osmolality were only present in the ICU group and few per patient. Dynamics of median sodium in all groups followed the same pattern with hyponatremia at admission and slowly increasing levels of sodium over the first weeks of hospitalization.

**Figure 2:**
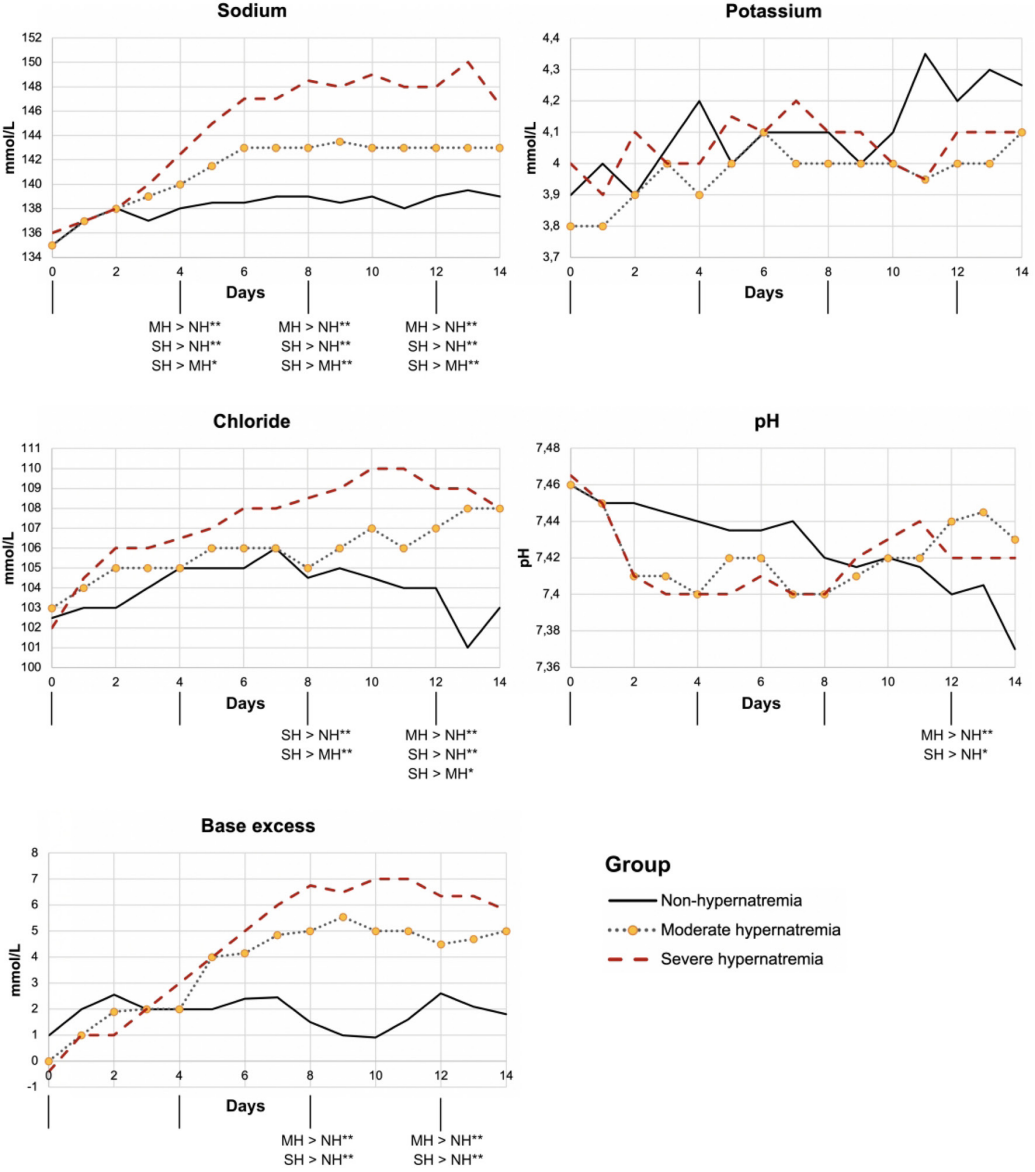
Laboratory parameter dynamics depending on peak level of sodium in the 223 critically ill patients. Dynamic pattern of laboratory parameters (sodium, potassium, chloride, pH, and base excess) the first 2 weeks of hospitalization, depending on the peak level of sodium. *P-value < 0.05. ^**^ *P*-value < 0.01. NH, non-hypernatremia; MH, moderate hypernatremia; SH, severe hypernatremia.

The different patterns of laboratory parameters of the ICU group, containing critically ill patients, divided into groups by peak sodium are presented in Fig. 2, and information about the number of patients contributing to each median is shown in Supplementary Table 3. On day 0, no differences between the groups were seen in any of the five parameters investigated (sodium, potassium, chloride, pH, and base excess). On day 4, only differences between sodium levels could be shown. On day 8, in additional to differences in sodium levels, there was a significant difference in chloride levels between the moderate hypernatremia group and the severe hypernatremia group as well as significant differences in base excess between the patients with no hypernatremia and the other two groups. On day 12, group ANOVA showed significant differences in all parameters except potassium. The non-hypernatremia group was different from all the other groups, and there were significant differences between moderate and severe regarding sodium and chloride. Potassium levels showed significant differences between the non-hypernatremia and moderate hypernatremia groups, but the group ANOVA was not significant (*P* = 0.052). For more information on group differences and *P*-values see Supplementary Table 4. Regarding serum osmolality, increasing levels during the course of hospitalization occurred with a median of 315 mOsmol/kg a week after admission.

The median critically ill patient in the group with non-hypernatremia was initially hyponatremic with respiratory alkalosis and levels of chloride and potassium in the lower range of normal. Over time, pH slowly decreased and the other parameters increased to the upper normal range. On admission, the hypernatremia groups also had a picture of hyponatremia, respiratory alkalosis, and levels of chloride and potassium in the lower range of normal. Shortly after admission, pH dropped to normal levels, and BE increased together with chloride to end up in metabolic alkalosis after 7–10 days. Compared to the moderate hypernatremia group, the severe hypernatremia group developed a more pronounced metabolic, hyperchloremic alkalosis.

The dynamics of electrolytes and acid-base parameters of all the critically ill patients are presented in Fig. 2. As seen in Supplementary Table 3, daily measurements were available for the majority of the critically ill, but not for all due to preanalytical/analytical issues, missed sampling, or deceased patients.

For the patients in need of intensive care, potassium substitution was very common, stated in the patient medical records. Therefore, a subgroup analysis (Kruskall–Wallis ANOVA) of potassium levels in the ward group was performed and found significant (*P* = 0.044). Pairwise comparison showed lower potassium in the group with severe hypernatremia compared to the group with non-hypernatremia (*P* = 0.024) (potassium at day of peak sodium).

### Relationships between hypernatremia, time in hospital, and the need for mechanical ventilation in the total cohort

The presence of hypernatremia was associated with a long time stay in the hospital (*P* < 0.001, Mann–Whitney *U*-test), with a median hospital stay of 22 days in the hypernatremia group and 10 days in the non-hypernatremia group. The need for ventilatory support was seen in a larger proportion (*P* < 0.001, Pearson’s *χ*^2^ test) of the hypernatremia group (144/170, 85%) compared to the non-hypernatremia group (47/236, 20%).

### Effects of hypernatremia on the likelihood of death in the total cohort

To investigate the effect of hypernatremia on the likelihood of death, a binary logistic regression was conducted. Age, gender, peak sodium level, and presence of diabetes mellitus type 2, hypertension, and OPD were entered into the model. The logistic regression model was statistically significant (*χ*^2^(6) = 80.737, *P* < 0.001). Three of the individual variables were statistically significant; age (*P* < 0.001), gender (*P* = 0.017), and the presence of hypernatremia. An increase in age by 1 year was associated with 1.075 (95% CI 1.048–1.104) times higher odds of death, and males had 2.336 (95% CI 1.162–4.696) times higher odds of death compared to females. Presence of hypernatremia was associated with 3.942 (95% CI 2.269–6.851) times higher odds of death. To further investigate possible contribution or association with volemic status, an additional model was run with the added covariate hematocrit on the day of the sodium peak. For the non-hypernatremia group, the day of median sodium peak in the hypernatremia group, day 9, was used. This model did not show any association between hematocrit (*P* = 0.488) and risk of death, but still a significant association between hypernatremia and death (*P* < 0.001). Higher sodium was also associated with a higher likelihood of death, as an increase by 1 mmol/L was associated with 1.087 (95% CI 1.034–1.143) times higher odds of death.

### Further analysis regarding volemic status

**Figure 3:**
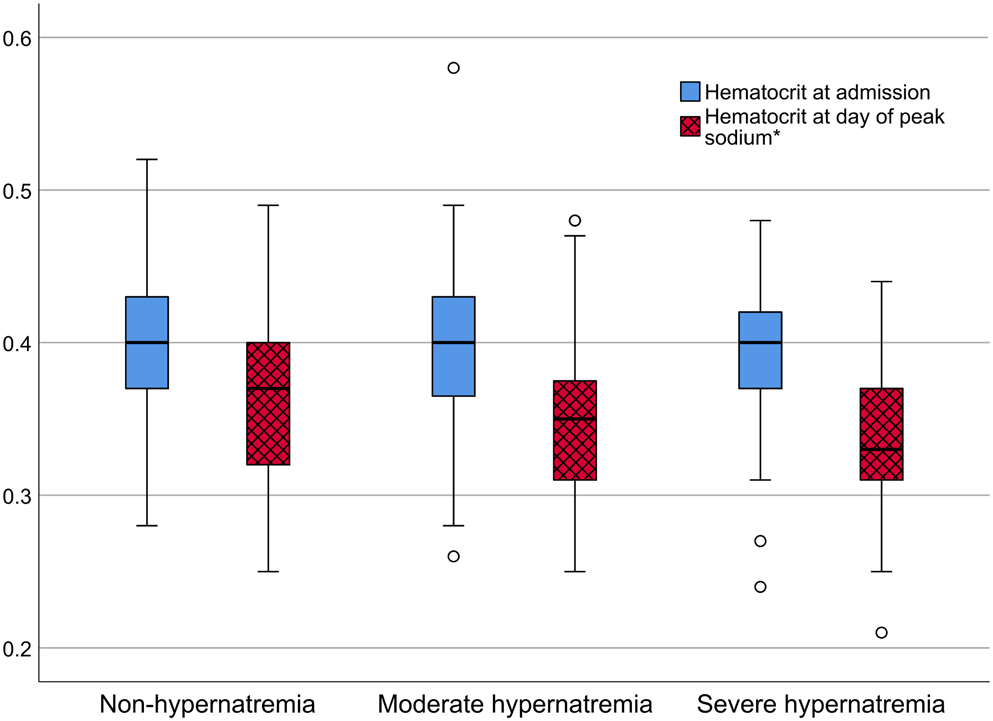
Hematocrit on admission and on the day of peak sodium of the whole cohort. Box plots with medians and interquartile range of hematocrit on admission and on the day of peak sodium.

There was no difference between the three groups in hematocrit on admission (*P* = 0.764). There was a significant difference in hematocrit between the groups on the day of sodium peak (*P* = 0.002 using one-way ANOVA). The median hematocrit levels decreased in the groups with higher sodium levels. We found a median value of 0.37 in the non-hypernatremia group, 0.35 in the moderate hypernatremia group, and 0.33 in the severe hypernatremia group. The hematocrit values in the moderate and severe hypernatremia groups are thus below reference levels on the day of peak sodium levels, consistent with hypervolemia or dilution (Fig. 3). In all groups, there was a significant decrease in hematocrit (*P* < 0.001 for all), as assessed by Wilcoxon signed ranks tests, between admission and the day of the sodium peak.

## Discussion

In this study, 42% of 406 patients with severe COVID-19 developed hypernatremia during the first 2 weeks of hospitalization. The patients with hypernatremia were admitted to the hospital for twice as long and had a higher risk of death than patients without hypernatremia, after adjusting for age, sex, comorbidities, and level of hematocrit on the day of sodium peak. Lower levels of sodium and potassium during admission were associated with the need for mechanical ventilation. Decreased pH at admission was associated with both death and the need for mechanical ventilation. The critically ill patients frequently developed a combination of hypernatremia, need for potassium substitution, metabolic alkalosis, and decreased levels of hematocrit.

Our finding of 57% of patients suffering from hyponatremia during admission is higher than previous studies, 25–52%, (2, 3, 5, 22) potentially because our cohort only included patients with severe COVID-19. Decreased levels of sodium during admission increased the odds of mechanical ventilation but were not associated with death. Most patients in the cohort had a very short time span between admission to the hospital and admission to ICU. However, even though hyponatremia was very common on the day of admission to the ICU, sodium levels increased over time, and hyponatremia was much less common at the time of death.

In atypical viral pneumonia with respiratory failure, such as COVID-19, SIADH is known to be one of the most common underlying causes of hyponatremia (30). In theory, patients presenting with hyponatremia during admission caused by SIADH could be of higher risk of developing severe hypernatremia later due to depletion of available AVP. This is difficult to substantiate due to patients seeking medical care at different times and possible individual variation in AVP storage pool. In addition, synthetic AVP (desmopressin) is used as procoagulant treatment, for example, in von Willebrand’s disease (vWD), as it increases platelet release of coagulation factor VIII and von Willebrand factor after administration. During admission when the patients presented with low levels of sodium and suspected SIADH, the potential high release of AVP could be a part of the procoagulant state of COVID-19 further impaired by hyperosmolality which also increases the risk of venous thromboembolism (31). Case reports of patients with COVID-19 and hyponatremia originating from both hypovolemia and SIADH, during admission, have recently been published (6, 10, 32).

The high incidence (42%) of in-hospital hypernatremia found in our cohort could be an viral effect on endocrine functions, physiological reactions to severe disease, and/or increased fluid loss compared to intake from iatrogenic dehydration caused by a negative fluid balance. Dehydration following treatment according to the acute respiratory distress syndrome (ARDS) protocol together with the virus disturbing endocrine functions, like the Renin-angiotensin-aldosterone system (RAAS), would be expected to shift the electrolyte pattern from hyponatremia to hypernatremia. It is notable that more ICU patients were receiving treatment for hypertension before the onset of COVID-19 in the group that did not develop hypernatremia, and it can be speculated that if the development of hypernatremia was inhibited by the anti-hypertensive drugs, possibly by modulation of the RAAS. Other possible contributing factors could be osmotic diuresis due to the severe catabolism seen in critically ill ICU patients (33).

In the present cohort, almost all patients with sodium levels above 149 mmol/L received active sodium-lowering treatment, according to the patient medical record. There was also a significant decrease in hematocrit in both groups with hypernatremia between admission and day of sodium peak, speaking against dehydration as a main contributor to the hypernatremia. Therefore, we believe that the very high sodium levels were unintended and likely caused by the disease.

In addition, there was an increased length of hospital stay, need for mechanical ventilation, and mortality in the hypernatremia group compared to the non-hypernatremia group. Altogether, these clinical observations suggest that hypernatremia is an indicator of severe illness.

Zimmer *et al.* reported a case series of six ICU patients with severe COVID-19 and hypernatremia (15). In their study, no obvious relationship betweenh sodium input and sodium levels was seen, information that is lacking in our study. Their patients suffered from hyperchloremia, a need for potassium replacement, and no sufficient lowering of sodium levels with standard management, as in our cohort. The clear similarities in biomarker patterns between our studies suggest a common underlying cause. In our study, potassium levels were difficult to assess from laboratory results due to the replacement of potassium in the ICU group, similar to the Zimmer *et al.* study. Despite this, we were able to show that patients with severe hypernatremia in the non-ICU group had significantly lower potassium levels than those without hypernatremia, and this together with information from the medical records describing potassium substitution in the ICU group indicates that patients with severe COVID-19 are likely to have a negative potassium balance.

Looking further into potential biomarker patterns caused by COVID-19, augmentation of BE is often a compensatory mechanism in respiratory acidosis, the body’s way of increasing low pH due to respiratory failure. In the current cohort, the pH was alkalotic in a majority of the patients during admission, and the respiratory alkalosis is suspected to be a result of the silent hypoxia, without hypercapnia, caused by viral pneumonia. Within a few days, pH decreased to normal range in the hypernatremia patients whereas the decrease was much slower in the non-hypernatremia group, most likely due to the high number of intubated patients in the hypernatremia groups. The rise of BE started during admission, in all groups, with peaking days from 7 to 11. The metabolic alkalosis was characterized as chloride resistant, or hyperchloremic, which could be indicating an effect of aldosterone and/or dehydration.

A limitation of this study is the lack of data on fluid input and output. In addition, the retrospective design of the study does not allow complete identification of the cause of the high frequency of hypernatremia. Potassium infusions in the ICU group made it difficult to assess the untreated levels of hypokalemia present.

Another issue is that hemolysis is not registered by the machines used for blood gas analysis to the same extent and way as routine biochemistry lab does which potentially leads to a risk of falsely elevated potassium levels. The presence of CRRT is also a potential source of error considering its effect on electrolyte levels. Due to the retrospective nature of the study, patients are not sampled according to a specific protocol leading to some variations in the timing of blood tests between the patients. This study, however, represents a large clinical consecutively recruited cohort of patients with severe COVID-19, adding to the generalizability of our results.

## Conclusion

Based on our findings from a large cohort of patients with severe COVID-19, hyponatremia during admission followed by the development of hypernatremia in the following weeks is common. Furthermore, patients with hypernatremia displayed a more severe course of COVID-19, and the mortality rate was higher in this group compared to the patients not developing hypernatremia. This makes hypernatremia a possible indicator of severe disease and highlights the importance of sodium monitoring.

Combined, our results show that patients with COVID-19 and hypernatremia also have a potassium deficiency, metabolic alkalosis, and hemodilution (decreased hematocrit). This may be explained by an over-activation of the RAAS, but to further understand the pathophysiology behind the variations in sodium levels in severe COVID-19, prospective studies with structured measurements of serum levels of angiotensin II, aldosterone, AVP, copeptin, urinary sodium, potassium, and osmolality, together with clinical information of fluid and sodium input and output, would be of great value.

## Supplementary Material

Supplementary table 1: Title of data: Laboratory parameter and equipment information

Supplementary table 2: Title of data: Corresponding number of samplings per day in figure 1

Supplementary table 3: Title of data: Corresponding number of patients in every calculated median in figure 2.

Supplementary table 4: Title of data: Differences between laboratory parameters in the groups (NH = non-hypernatremia, MH = moderate hypernatremia, SH = severe hypernatremia).

## Declaration of interest

The authors declare that there is no conflict of interest that could be perceived as prejudicing the impartiality of the research reported.

## Funding

This research did not receive any specific grant from any funding agency in the public, commercial or not-for-profit sector.

## Author contribution statement

A S designed the study, collected, analyzed and interpreted the data, wrote the first draft, and revised the manuscript. S R has interpreted the data and revised the manuscript. H S analyzed and interpreted the data, and revised the manuscript. C H has supervised the course of the study, interpreted the data, and revised the manuscript. All authors read and approved the final manuscript.
