# Supplementary material for: Electrolyte and acid-base imbalance in severe COVID-19
Source: Endocr Connect. 2021 Jun 22;10(7):805–14. doi: 10.1530/EC-21-0265 (PMC8346182; doi:10.1530/EC-21-0265)
Supplement: Supplementary table 1: Title of data: Laboratory parameter and equipment information [file supplementary_table_1.pdf]

Supplementary table 1:

Title of data: Laboratory parameter and equipment information

Description of data: Table of properties of analytes and their corresponding measurement equipment.

Analyte	Equipment	Manufacteurer	Method	Level/CV%	Reference range	Unit
Sodium (Plasma or arterial blood)	Cobas 8000 or ABL800 flex	Roche Diagnostics, Switzerland or Radiometer, Denmark	Ion sensitive electrode, indirect method or Ion sensitive electrode, direct method	140/3%<br>160/3%	137-144	mmol/L
Potassium (Plasma or arterial blood)	Cobas 8000 or ABL800 flex	Roche Diagnostics, Switzerland or Radiometer, Denmark	Ion sensitive electrode, indirect method or Ion sensitive electrode, direct method	4/3%<br>7/3%	3,5-4,6	mmol/L
Chloride (Plasma or arterial blood)	Cobas 8000 or ABL800 flex	Roche Diagnostics, Switzerland or Radiometer, Denmark	Ion sensitive electrode, indirect method or Ion sensitive electrode, direct method	100/3%<br>115/3%	98-107	mmol/L
pH (Arterial blood)	ABL800 flex	Radiometer, Denmark	Potentiometry	6,8 / 0,15%<br>7,1 / 0,15%<br>7,4 / 0,15%<br>7,6 / 0,15%	7,35-7,45	
Base excess (Arterial blood)	ABL800 flex	Radiometer, Denmark	Calculated from other parameters	Not applicable	-3 – 3	mmol/L
Osmolality (Serum)	Osmometer	Advanced instruments, United states	Freezing point depression	280/3%	280-300	mosmol/kg
Hematocrite (Whole blood)	Sysmex XN-9000	Sysmex Europe, Germany	Impedance	0,17 / 3%<br>0,35 / 3 %	F: 0,35-0,46<br>M: 0,39-0,50	fraction
Creatinine	Cobas 8000	Roche Diagnostics,	Enzymatic reaction,	70 / 7%	F <90	µmol/L

(Plasma)		Switzerland	photometry	560 / 4 %	M <100	
