# Supplementary material for: Electrolyte and acid-base imbalance in severe COVID-19
Source: Endocr Connect. 2021 Jun 22;10(7):805–14. doi: 10.1530/EC-21-0265 (PMC8346182; doi:10.1530/EC-21-0265)
Supplement: Supplementary table 2: Title of data: Corresponding number of samplings per day in figure 1 [file supplementary_table_2.pdf]

Supplementary table 2:

Title of data: Corresponding number of samplings per day in figure 1

Description of data: Table of number of samplings per day in figure 1

**Number of patients forming medians**															
**Analyte**	**Day 0**	**Day 1**	**Day 2**	**Day 3**	**Day 4**	**Day 5**	**Day 6**	**Day 7**	**Day 8**	**Day 9**	**Day 10**	**Day 11**	**Day 12**	**Day 13**	**Day 14**
**Sodium, n**	374	339	327	316	299	282	272	249	247	223	201	194	177	167	118
**Potassium, n**	362	335	324	321	296	283	266	252	241	223	205	192	180	165	150
