# Supplementary material for: Electrolyte and acid-base imbalance in severe COVID-19
Source: Endocr Connect. 2021 Jun 22;10(7):805–14. doi: 10.1530/EC-21-0265 (PMC8346182; doi:10.1530/EC-21-0265)
Supplement: Supplementary table 3: Title of data: Corresponding number of patients in every calculated median in figure 2. [file supplementary_table_3.pdf]

Supplementary table 3:

Title of data: Corresponding number of patients in every calculated median in figure 2.

Description of data: Number of patients in each median in Figure 2 (HN = hypernatremia) day 0-14 of hospitalization.

		Day 0	1	2	3	4	5	6	7	8	9	10	11	12	13	14
Sodium	*Non-HN*	73	62	62	58	61	56	54	58	57	54	45	41	41	42	25
	Moderate HN	85	79	75	83	80	82	80	82	79	74	72	70	67	65	49
	Severe HN	50	45	44	46	46	49	48	48	48	47	48	45	44	41	30
Potassium	*Non-HN*	72	62	62	62	60	54	55	57	57	53	47	40	43	44	34
	Moderate HN	86	78	74	82	79	82	79	81	79	75	72	70	67	65	57
	Severe HN	38	35	41	45	45	46	47	46	45	44	45	40	41	37	39
Chloride	*Non-HN*	65	44	50	46	45	45	47	48	44	43	38	33	30	30	21
	Moderate HN	61	57	63	74	75	78	76	75	76	68	66	64	62	57	54
	Severe HN	41	35	39	44	42	48	46	47	46	45	47	45	43	38	39
pH	*Non-HN*	66	46	51	46	45	46	46	47	45	42	37	34	31	32	25
	Moderate HN	72	58	64	72	76	77	78	76	76	70	67	64	62	58	52
	Severe HN	50	34	40	45	45	49	47	48	48	46	48	43	43	38	35
Base excess	*Non-HN*	50	44	48	44	45	44	45	48	46	44	36	35	31	32	23
	Moderate HN	53	59	63	73	76	78	78	76	76	70	67	65	63	59	51
	Severe HN	30	31	39	44	44	47	46	46	46	45	47	43	42	38	38
