# Supplementary material for: Electrolyte and acid-base imbalance in severe COVID-19
Source: Endocr Connect. 2021 Jun 22;10(7):805–14. doi: 10.1530/EC-21-0265 (PMC8346182; doi:10.1530/EC-21-0265)
Supplement: Supplementary table 4: Title of data: Differences between laboratory parameters in the groups (NH = non-hypernatremia, MH = moderate hypernatremia, SH = severe hypernatremia). [file supplementary_table_4.pdf]

Supplementary table 4:

Title of data: Differences between laboratory parameters in the groups (NH = non-hypernatremia, MH = moderate hypernatremia, SH = severe hypernatremia).

Description of data: Table of p- values from group ANOVA and pairwise comparison.

**Group differences**				
	**Day 0**	**Day 4**	**Day 8**	**Day 12**
**Sodium**				
Group ANOVA	p = 0.996	**p < 0.001**	**p < 0.001**	**p < 0.001**
NH vs MH	p = 0.936	**p = 0.001**	**p < 0.001**	**p < 0.001**
NH vs SH	p = 0.946	**p < 0.001**	**p < 0.001**	**p < 0.001**
MH vs SH	p = 0.962	**p = 0.024**	**p < 0.001**	**p < 0.001**
**Potassium**				
Group ANOVA	p = 0.282	p = 0.168	p = 0.974	p =0.052
NH vs MH	p = 0.135	p = 0.097	p = 0.842	**p = 0.021**
NH vs SH	p = 0.650	p = 0.109	p = 0.975	p = 0.149
MH vs SH	p = 0.297	p = 0.861	p = 0.858	p = 0.309
**Chloride**				
Group ANOVA	p = 0.538	p = 0.138	**p < 0.001**	**p < 0.001**
NH vs MH	p = 0.293	p = 0.488	p = 0.249	**p = 0.001**
NH vs SH	p = 0.425	p = 0.051	**p < 0.001**	**p < 0.001**
MH vs SH	p = 0.975	p = 0.144	**p = 0.002**	**p = 0.013**
**pH**				
Group ANOVA	p = 0.317	p = 0.280	p = 0.868	**p = 0.021**
NH vs MH	p = 0.521	p = 0.199	p = 0.693	**p = 0.006**
NH vs SH	p = 0.133	p = 0.165	p = 0.811	**p = 0.038**

MH vs SH	p = 0.337	p = 0.507	p = 0.640	p = 0.599
**Base excess**				
Group ANOVA	p = 0.299	p = 0.287	**p < 0.001**	**p = 0.002**
NH vs MH	p = 0.296	p = 0.146	**p < 0.001**	**p = 0.004**
NH vs SH	p = 0.122	p = 0.186	**p < 0.001**	**p = 0.001**
MH vs SH	p = 0.641	p = 0.987	p = 0.259	p = 0.249
